# Evaluation of Serum Antioxidant Activity in Type 2 Diabetes and Prediabetes: Links with Nutritional and Anthropometric Factors—Preliminary Studies

**DOI:** 10.3390/cimb47121017

**Published:** 2025-12-05

**Authors:** Michalina Banaszak, Grzegorz Kosewski, Ilona Górna, Sławomira Drzymała-Czyż

**Affiliations:** 1 Poznan University of Medical Sciences, Department of Bromatology, Rokietnicka 3, 60-806 Poznan, Poland; michalina.banaszak@student.ump.edu.pl (M.B.); grzegorzkosewski@ump.edu.pl (G.K.); drzymala@ump.edu.pl (S.D.-C.); 2 Poznan University of Medical Sciences, Doctoral School, Bukowska 70, 60-812 Poznan, Poland

**Keywords:** total antioxidant capacity, ABTS, DPPH, FRAP, metformin

## Abstract

Background: Type 2 diabetes (T2DM) and prediabetes are growing public health problems worldwide. Oxidative stress plays a key role in the pathogenesis and progression of carbohydrate metabolism disorders. Metformin is an antidiabetic drug that significantly affects the oxidative-antioxidant balance. This study aimed to compare serum total antioxidant capacity (TAC) in individuals with T2DM, prediabetes, and healthy controls, and to assess the impact of dietary factors and metformin treatment on antioxidant parameters. Methods: The study involved 49 adults (aged 40–70 years) assigned to three groups: those with T2DM (*n* = 19), those with prediabetes (*n* = 12), and healthy controls (*n* = 18). Serum TAC was assessed using three spectrophotometric assays: DPPH, ABTS, and FRAP. A nutritional assessment was performed based on a three-day dietary recall, analysed using DietetykPro software. Statistical analyses included Kruskal–Wallis tests with post hoc corrections and Spearman correlation. Results: The prediabetes group demonstrated the lowest TAC values across all tests, while individuals with T2DM demonstrated higher levels using the ABTS and FRAP tests, which may reflect group-specific factors such as treatment or metabolic regulation. The differences between groups showed moderate to large effect sizes, including η^2^ = 0.24 for ABTS, η^2^ = 0.14 for DPPH and η^2^ = 0.13 for FRAP, indicating biologically meaningful alterations in antioxidant capacity. Negative correlations were observed between antioxidant activity, as measured by the DPPH test, and body weight (*p* = 0.0095) and BMI (*p* = 0.0381), indicating that increased body weight may impair serum antioxidant capacity. After applying the FDR correction, significant correlations were observed between ABTS values and vitamin B5 (*p* = 0.0004, q = 0.0135), omega-6 (*p* = 0.0042, q = 0.0220), phosphorus (*p* = 0.0009, q = 0.0328), calcium (*p* = 0.0024, q = 0.0176) and zinc (*p* = 0.0012, q = 0.0138) intake. Other associations with anthropometric and dietary variables were observed as non-significant trends. Conclusions: The prediabetes group exhibited lower TAC, indicating a redox profile that differs from both healthy individuals and those with T2DM. Dietary quality, including adequate intake of selenium, could support antioxidant defence mechanisms, whereas excess body weight and high intake of omega-6 may impair them. The results also suggest that metformin may modulate TAC, supporting adaptive responses to oxidative stress in T2DM. These findings highlight the potential importance of dietary and pharmacological interventions in maintaining oxidative-antioxidant balance in metabolic disorders.

## 1. Introduction

Type 2 diabetes (T2DM) is a significant problem today—up to 500 million people worldwide suffer from this disease, and this number could rise to 783 million by 2045 [1,2,3]. It is a metabolic disorder characterised by high blood glucose levels (hyperglycaemia) caused by inadequate insulin action in the blood—insulin resistance. Long-term hyperglycaemia is associated with a number of complications, including retinopathy, nephropathy, neuropathy, and micro- and macroangiopathy. People at particular risk of developing T2DM are those with prediabetes, overweight, or obesity (BMI ≥ 25 kg/m^2^ and/or waist circumference ≥80 cm in women or ≥94 cm in men), a family history of diabetes, low physical activity, and other lifestyle diseases, including dyslipidaemia, hypertension, and cardiovascular disorders [4]. When blood glucose levels exceed 100 mg/dL, we speak of prediabetes [5], which can last up to 10 years before the onset of full-blown T2DM [6,7].

The development of T2DM is a multifactorial process. As excess body weight develops, muscles, liver, and adipose tissue become less sensitive to insulin, resulting in impaired glucose uptake from the bloodstream [8,9,10]. Furthermore, excessive lipolysis in adipose tissue leads to increased free fatty acid concentrations (lipotoxicity), which translates into impaired glucose uptake by muscles and increased insulin resistance [11]. Furthermore, pancreatic β-cells initially increase insulin production (hyperinsulinemia), but over time, their function deteriorates. Reduced insulin production manifests as increased blood glucose levels, ultimately leading to the diagnosis of T2DM [8,9,12].

Oxidative stress plays a key role in T2DM, leading to a self-perpetuating vicious cycle. It is defined as an imbalance between oxidants (free radicals, reactive oxygen species—ROS) and antioxidants. Free radicals and ROS are natural byproducts of metabolic processes, but in excess, they become harmful to the body, leading to impaired redox signalling and cellular dysfunction [13,14,15]. Hyperglycaemia leads to excessive production of ROS, which damages proteins, lipids, and DNA and impairs insulin signalling and β-cell function. These processes exacerbate insulin resistance, leading to increased blood glucose levels [16,17,18]. Furthermore, oxidative stress activates inflammatory pathways, resulting in increased production of inflammatory cytokines and ROS. Therefore, chronic, low-grade inflammation is observed in the course of T2DM [16,19].

It is important to remember that individually tailored pharmacological treatment in the multifactorial treatment of T2DM (in addition to treating comorbidities) is crucial in preventing and slowing the progression of chronic diabetes complications. Lifestyle modification, consisting of weight loss (by reducing the caloric intake of the diet) and incorporating physical activity, is an integral part of this approach. For most patients with T2DM, the first-line treatment is metformin, a biguanide. Metformin has numerous beneficial effects, including reduced hepatic glucose production, increased peripheral insulin sensitivity, and a positive impact on the lipid profile. It does not cause hypoglycaemia, which may be important in older adults, and has minimal side effects (gastrointestinal disturbances) [4]. In addition to its glucose-lowering effects, metformin has a number of additional benefits, including antioxidant and anti-inflammatory properties. Studies show that metformin can reduce the production of reactive oxygen species (ROS) (by inhibiting mitochondrial complex I and reducing NADPH oxidase expression), increase the expression of antioxidant enzymes (including superoxide dismutase, catalase, and glutathione peroxidase) via the AMPK and Nrf2 pathways, and protect against oxidative damage [20,21,22,23,24,25].

Based on literature reports, the study objective was to assess differences in serum antioxidant activity, measured by DPPH, ABTS, and FRAP assays, between healthy individuals, individuals with prediabetes, and individuals with T2DM, and to determine the role of metformin and dietary patterns as potential factors that modify antioxidant activity. This was a preliminary study.

## 2. Materials and Methods

### 2.1. Materials

Forty-nine individuals meeting the inclusion criteria for each group were recruited for the study. The first study group (T2DMG) (*n* = 19) consisted of individuals diagnosed with T2DM (according to WHO criteria [26], T2DM duration time: 10.59 ± 6.90), aged 40–70 years, who were treated with a single antidiabetic medication and were free from diabetic complications. The second study group (PG) (*n* = 12) comprised individuals aged 40–70 years with prediabetes, as determined by fasting glucose measurements within the range of 100–125 mg/dL. Participants in this group did not take hypoglycaemic medications or insulin therapy. The control group (CG) (*n* = 18) comprised healthy individuals aged 40–70 years. The same exclusion criteria applied to each group: treatment with insulin or multiple oral antidiabetic medications (in the case of the T2DMG), as well as cancer, autoimmune diseases, and alcohol/drug addiction. Participants were recruited for the study during the preventive campaign “Biała Sobota” at the District Hospital in Czarnków, taking place from January to July 2025. During the campaign, participants received medical and dietary advice, and anthropometric measurements (height and weight) were taken. They were also asked to complete a personal questionnaire and a nutritional interview. Participation in the study was voluntary. Before participating in the experiment, all participants signed an informed consent form in accordance with the principles outlined in the Declaration of Helsinki. All research was conducted with the approval of the Bioethics Committee of the Poznań University of Medical Sciences—No. 854/22.

### 2.2. Methods

#### 2.2.1. Dietary Assessment

Dietary assessment was conducted based on a 3-day dietary interview and then analysed using DietetykPro software (https://dietetykpro.pl, accessed on 3 September 2025) (Wrocław, Poland). The obtained results enabled the determination of the selected nutrient content. Nutrient intake was assessed in relation to established dietary standards for the given population.

#### 2.2.2. Blood Collection

The study material consisted of venous blood collected from participants in the morning, on an empty stomach (at least 12 h after their last meal), using vacuum sets. Qualified nurses collected blood samples. After collection, the samples were stored under sterile conditions. After clotting, the blood was centrifuged within one hour at 2500 rpm, and the resulting serum was used for testing. The biological material was stored immediately in a freezer at −80 °C until it was tested. Each batch was thawed a maximum of two times, and the analyses were performed within one month of collection. Samples showing haemolysis were excluded from analysis.

#### 2.2.3. Total Antioxidant Capacity (TAC) Determination

To determine the antioxidant potential, free radical reduction methods were used, using the DPPH (2,2-diphenyl-1-picrylhydrazyl radical) radical and the ABTS (2,2′-azino-bis(3-ethylbenzothiazoline-6-sulfonic acid)) cation radical, as well as the Ferric Ion Reducing Antioxidant Parameter (FRAP) method for reducing iron Fe^3+^ to Fe^2+^. All blood samples were analysed in duplicate to ensure the reliability and reproducibility of the measurements.

#### 2.2.4. DPPH Assay

Antioxidant capacity was determined using the DPPH assay described by Norma et al. [27]. Briefly, 0.8 mL of the DPPH radical solution in methanol (0.1 mM) was mixed with 0.78 mL of phosphate buffer (0.01 M, pH 7.4) and 0.02 mL of serum. The mixture was incubated in the dark for 30 min at room temperature, after which the absorbance was measured at λ = 517 nm (Analytik Jena Spekol 1500, Jena, Germany). The serum’s ability to scavenge free radicals was expressed as Trolox equivalent antioxidant capacity (TEAC). TEAC values were calculated from the Trolox equivalent (TE) calibration curve (y = 975.77x + 1.3243, R^2^ = 0.97). Results were expressed as micromoles of Trolox equivalents per millilitre of serum (µmol TE/mL). Percent inhibition of the DPPH radical (based on the formula presented above) was presented alongside the Trolox equivalent antioxidant capacity (TEAC) in the results table.

#### 2.2.5. ABTS Assay

Antioxidant capacity was determined using the ABTS assay described by Miller et al. [28]. Briefly, 2 mL of a methanolic solution of the ABTS radical cation (7 mM) and 0.01 mL of serum were incubated in the dark for 30 min. Absorbance was then measured at a wavelength of λ = 734 nm (Analytik Jena Spekol 1500, Jena, Germany). The serum’s free radical scavenging capacity was expressed as TEAC. TEAC values were calculated from the TE calibration curve (y = 2292.3x + 6.1557, R^2^ = 0.97). Results are expressed as micromoles of Trolox equivalents per millilitre of serum (µmol TE/mL).

#### 2.2.6. FRAP Assay

The antioxidant capacity of the serum was determined using the FRAP assay, as described by Benzie and Strain [29], with minor modifications. Briefly, 2.4 mL of FRAP reagent, consisting of 100 mL of acetate buffer (10 mM, pH 3.6), 10 mL 2,4,6-Tri(2-pyridyl)-1,3,5-triazine (TPTZ) and 10 mL of iron(III) chloride solution (20 mM), was incubated with 0.08 mL of serum for 2 min in a water bath at 37 °C. After incubation, the absorbance was measured at a wavelength of λ = 595 nm (Analytik Jena Spekol 1500, Jena, Germany). The reducing power of the serum was determined from a calibration curve prepared from a solution of iron(II) metal sulphate (y = 3.1182x + 0.3109, R^2^ = 0.99). From the equation of the calibration curve, the corresponding Fe^2+^ ion concentration was calculated and expressed as micromoles of Fe^2+^ per millilitre of serum (µmol Fe^2+^/mL).

#### 2.2.7. Statistical Analysis

This study was designed as a preliminary study. The sample size was determined based on feasibility considerations rather than formal power calculations. Given the exploratory nature of the study, a higher margin of error (14%) was accepted for estimating population parameters. This approach allowed for initial assessment of trends in the measured outcomes, while acknowledging that the study is not sufficiently powered to detect small to moderate effects.

Statistical analysis was performed using PQStat v.1.8.4 (PQStat Software, Poznań, Poland). Normality of data distribution was assessed using the Shapiro–Wilk test. Due to the lack of normality for most of the analysed variables, results were presented as medians (Me) and interquartile ranges (IQRs) and means (X) ± standard deviation (SD) to facilitate interpretation and to allow comparison with previous literature, although medians and IQRs should be considered the primary indicators. The nonparametric Kruskal–Wallis test was used for comparisons between the three groups (CG—healthy individuals, PDG—individuals with prediabetes, T2DMG—individuals with T2DM), with Dunn’s post hoc test for nonparametric data. Qualitative variables (gender, place of residence, number of medications taken) were compared between groups using Fisher’s exact test. The relationships between antioxidant activity parameters (DPPH, ABTS, FRAP) and selected dietary variables and sociodemographic factors were assessed using Pearson’s correlation coefficient (r) for normally distributed data or Spearman’s correlation coefficient (ρ) for nonparametric data. Due to the small size of each group, correlation analysis was performed on the entire study population (*n* = 49), allowing for the assessment of overall relationships between dietary variables, sociodemographic factors, and total antioxidant capacity. However, it should be emphasised that this approach may mask intergroup differences, so the obtained results should be interpreted with caution. For correlation analyses involving more than 30 variables, q-values were calculated using the Benjamini–Hochberg false discovery rate (FDR) procedure to correct for multiple comparisons. Correlations with q < 0.05 were considered statistically significant, whereas correlations that did not reach this threshold or were calculated on smaller sets of variables are described as non-significant trends. It should also be noted that some dietary variables showed relatively high standard deviations in relation to the mean. This reflects the substantial inter-individual variability and the typically skewed distribution of dietary intake data, rather than data entry or calculation errors.

In addition to the *p*-value, the two-sided 95% confidence interval (95% CI) and the effect size measure, calculated as the partial eta-squared coefficient (η^2^), suitable for comparisons between the three groups in a nonparametric model, are also presented. η^2^ values were interpreted according to Cohen’s thresholds: 0.01 = small, 0.06 = moderate, and 0.14 = large. All tests were considered statistically significant at a significance level of *p* < 0.05.

## 3. Results

In the study, women predominated in all groups (75–89%) (*p* = 0.6480). The mean age of participants did not differ significantly between groups (*p* = 0.16), but the groups differed in terms of body weight, body mass index (BMI), and height. The highest body weight and BMI values were found in the T2DMG and PDG, the lowest in the CG (*p* < 0.001), and height was most significant in the CG (*p* = 0.005). Place of residence also differed significantly between groups (*p* = 0.0004). Mean fasting glucose and HbA_1c_ values were higher in the T2DMG than in the PDG (*p* = 0.0021 and *p* = 0.0013). The number of medications and supplements taken was significantly higher in the T2DMG and PDG compared with the CG (*p* = 0.009). Smoking, alcohol intake and physical activity did not differ between groups (*p* = 0.5579, 0.1987, 0.4710, respectively) (Table 1).

Sensitivity analyses comparing participants with T2DM who took metformin (*n* = 17) with those not receiving metformin (*n* = 30) showed significantly higher ABTS values in the metformin users (Mann–Whitney U = 178.5, *p* = 0.0372, Cohen’s d = 0.6206). Differences in FRAP (*p* = 0.0519) and DPPH (*p* = 0.1899) were not statistically significant, although effect sizes were moderate (d = 0.38–0.58).

**Figure 1:**
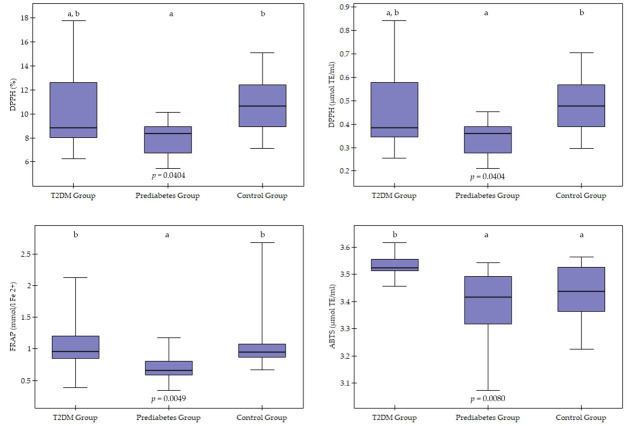
Graphic visualisation of antioxidant activity using the DPPH (2,2-diphenyl-1-picrylhydrazyl radical), ABTS (2,2′-azino-bis(3-ethylbenzothiazoline-6-sulfonic acid)) and FRAP (Ferric Reducing Antioxidant Power) methods in the study groups. T2DM—Type 2 Diabetes. Values are presented as median and interquartile range. Group differences were visualised using boxplots showing the median (horizontal line) and interquartile range (box), with whiskers representing 1.5× IQR. Differences between groups were assessed using the Kruskal–Wallis test with Dunn’s post hoc analysis (Bonferroni correction). Groups marked with different letters differ statistically significantly (e.g., a, b) (*p* < 0.05), whereas groups marked with the same letter do not differ statistically (egg, a, a or b, b) (*p* > 0.05).

In the study groups, antioxidant activity was determined by the DPPH, ABTS, and FRAP assays (Table 2, Figure 1). Serum antioxidant activity, as measured by the DPPH assay, differed significantly between the groups (*p* = 0.0404). Dunn’s post hoc analysis showed that the PDG had significantly lower DPPH assay values compared to the CG (*p* = 0.0339), whereas the T2DMG did not differ significantly from the other groups (*p* > 0.05).

Significant differences were found between groups using the ABTS assay (*p* = 0.0080). Individuals in the T2DMG had significantly higher antioxidant activity values using the ABTS assay than those in the PDG (*p* = 0.0189) and the CG (*p* = 0.0461). No significant differences were observed between PDG and CG (*p* > 0.05).

Antioxidant activity measured by the FRAP method also showed significant differences between groups (*p* = 0.0049). The lowest FRAP values were observed in the PDG, which were significantly lower than in the T2DMG (*p* = 0.0134) and the CG (*p* = 0.0083).

In addition to statistically significant differences between groups, moderate to large effect sizes were also observed (η^2^ = 0.13–0.24). The largest effect size was observed for antioxidant capacity determined by the ABTS method (η^2^ = 0.24), indicating clear differences between the studied metabolic groups. For DPPH parameters, the effect was moderate (η^2^ = 0.14–0.15), while for FRAP, the effect size was close to moderate (η^2^ = 0.128).

Dietary status was also assessed in the study groups, with the results presented in Table 3.

Analysis of energy value and macro- and micronutrient intake between groups (T2DMG, PDG, CG) revealed no significant differences in energy intake (*p* = 0.062), percentage of energy from fat (*p* = 0.434), and carbohydrates (*p* = 0.276). However, the proportion of protein in the diet was significantly higher in the T2DMG (22.2%) compared to the PDG (17.6%) and CG (14.6%) (*p* < 0.001). Differences also occurred in total protein intake, which was highest in T2DMG, intermediate in PDG, and lowest in CG (*p* = 0.004). In turn, the proportion of plant protein was significantly higher in the CG (*p* < 0.001), while animal protein did not differ significantly between groups (*p* = 0.082). There were no significant differences in total fat intake (*p* = 0.138), but fat quality differed significantly between groups. The T2DMG consumed the least monounsaturated fatty acids (MUFA) and polyunsaturated fatty acids (PUFA) and the most saturated fat compared to the PDG and CG. The CG, on the other hand, had the highest intake of MUFA, PUFA, omega-3, and omega-6 (*p* ≤ 0.007). Cholesterol intake was also highest in the CG (*p* = 0.007). The percentage of energy from carbohydrates in the diet was similar across all groups (*p* = 0.276), although in absolute terms, intake was lowest in the T2DMG and highest in the CG (*p* = 0.003). Simple sugar intake was significantly higher in the PDG and CG compared to the T2DMG (*p* = 0.003), whereas dietary fibre content was similar across all groups, ranging from ~18 to 20 g/d (*p* = 0.409).

Analysis of vitamin intake revealed significant differences for B vitamins (B2, B5, B6, B12; *p* ≤ 0.03), vitamin C (*p* = 0.004), and vitamin D (*p* = 0.03). The T2DMG had lower intakes of vitamins D and B5 compared to the CG, but higher intakes of vitamin C compared to the CG. Vitamin E, niacin, and folic acid intake did not differ significantly between groups.

Among minerals, significant differences were observed between groups in the intake of magnesium, zinc, phosphorus, iron, calcium, sodium, copper, and selenium (*p* ≤ 0.007). Most of the minerals mentioned were consumed in the highest amounts by the CG, while the T2DMG consumed them in the lowest amounts. The PDG generally achieved values intermediate between the T2DMG and CG.

Additional analysis indicated correlations between serum antioxidant activity measurements, conducted using various methods, and anthropometric parameters, as well as the level of individual nutrient intake (Table 4).

The relationships between antioxidant activity, as measured by the DPPH assay, and selected clinical parameters were assessed using the Spearman rank correlation coefficient. A significant negative correlation was found between TAC measured by the DPPH assay and body weight (r = −0.40, *p* = 0.0095) and BMI (r = −0.33, *p* = 0.0381), indicating that higher body weight and BMI were associated with lower antioxidant activity. Because these analyses involved only a small number of variables, no multiple-testing correction was applied. Furthermore, significant negative trends were found between antioxidant capacity, as measured by the DPPH assay, and cholesterol intake (r = −0.347, *p* = 0.026). However, this result was not significant after correction for multiple comparisons (q = 0.3332).

Further analysis revealed significant correlations between antioxidant capacity, as measured by the ABTS assay, and the level of intake of selected dietary components. A positive correlation was observed for pantothenic acid (vitamin B5, r = 0.53, *p* = 0.0004). A positive, although slightly weaker, relationship was also observed for selenium (r = 0.38, *p* = 0.0153) and the percentage of protein in the diet (r = 0.34, *p* = 0.0305). On the other hand, significant negative correlations were found for some fatty acids and minerals. Both the level of intake of omega-3 (r = −0.37, *p* = 0.0163) and omega-6 (r = −0.44, *p* = 0.0042) fatty acids, as well as monounsaturated (MUFA, r = −0.35, *p* = 0.0229) and polyunsaturated (PUFA, r = −0.35, *p* = 0.0239) fatty acids, showed a negative association with TAC according to the ABTS test. Considering minerals, significant negative correlations were observed for the intake of phosphorus (r = −0.50, *p* = 0.0009), calcium (r = −0.46, *p* = 0.0024), and zinc (r = −0.49, *p* = 0.0012). After applying the Benjamini–Hochberg correction for multiple comparisons, statistically significant relationships remained for vitamin B5 (q = 0.0135), omega-6 (q = 0.0220), phosphorus (q = 0.0328), calcium (q = 0.0176) and zinc (q = 0.0138). The associations with selenium, protein intake, omega-3, MUFA and PUFA did not remain significant after FDR correction and should therefore be interpreted as non-significant trends.

Significant negative correlations were observed between antioxidant capacity according to the FRAP method and the intake of omega-3 fatty acids (r = −0.353; *p* = 0.0130) and omega-6 fatty acids (r = −0.332; *p* = 0.0198). In contrast, no significant correlations were found between FRAP and the intake of antioxidant vitamins (C, E) or other nutrients. However, after applying the FDR correction for multiple testing, none of the correlations remained statistically significant (q > 0.05), indicating that these trends should be interpreted with caution.

No significant correlations were observed between the values of antioxidant capacity using the DPPH and ABTS radical assays and the FRAP method and anthropometric and biochemical parameters, as well as age, glucose concentration, HbA_1c_, smoking, alcohol intake, physical activity and other macro- and micronutrients.

## 4. Discussion

### 4.1. Analysis of the Usefulness of Oxidative Stress Assessment Methods

TAC assessment is a crucial element in oxidative stress research, facilitating an integrated evaluation of the balance between pro- and antioxidant substances. In the present study, TAC was assessed using three independent methods—DPPH, ABTS, and FRAP—which differ in both their operating principle and sensitivity to individual antioxidant classes (Table 2).

The DPPH assay, despite its simplicity, sensitivity, and good reproducibility, has limited usefulness in biological samples due to interference from plasma components and sensitivity to reaction conditions (pH, solvent, incubation time) [30]. In the case of biological samples such as serum or plasma, the DPPH assay faces additional limitations related to the presence of proteins, lipids, and other components that may interfere with the reaction [31].

Nevertheless, numerous studies use the DPPH method to assess TAC in plasma and serum. Studies in a population of 60 elderly patients with varying health statuses showed that DPPH values were 26.3% and FRAP values were 1.19 mmol FeCl2/L [32]. Another study conducted in a group of healthy, non-smoking men reported lower DPPH values (11.2%) and FRAP (382.0 mmol/L) [30]. Furthermore, this method assesses changes in the antioxidant capacity of plasma or serum after consuming products rich in phenolic compounds, such as apple juice [30]. For example, in the study by Gheflati et al. [33], which included 70 participants with T2DM, eight weeks of apple cider vinegar supplementation resulted in a significant increase in antioxidant capacity, as measured by the DPPH method, in the intervention group, suggesting an improvement in the body’s redox status.

The variation in the obtained results may be due to methodological differences, the participants’ health status, age, and dietary intake of antioxidant compounds, which underscores the difficulty in directly comparing literature data. Janaszewska et al. [34] emphasise that the DPPH test requires strictly controlled and repeatable reaction conditions. Similar limitations apply to the ABTS method, where too short an incubation time can lead to an incomplete reaction with thiols, while too long can lead to an excessive participation of amino acid residues (tyrosine, tryptophan), resulting in an overestimation of the TAC value. In the case of the FRAP method, the impact of these factors is less significant; however, caution should still be exercised when interpreting the results.

While the DPPH test should be used with caution in biological samples, the ABTS method is commonly employed to assess TAC, and numerous studies have highlighted its correlation with other antioxidant markers. A comparison of the DPPH, ABTS, and FRAP methods showed that the ABTS test was the most optimal method for assessing serum antioxidant activity, especially in population-based and clinical studies [34,35]. The reaction mechanism of the ABTS assay is based on both electron and hydrogen transfer, thus better reflecting TAC in complex biological matrices. [36] A study of 160 individuals with diabetes demonstrated that combination therapy (glucagon-like peptide-1 receptor agonist + sodium-glucose cotransporter-2 inhibitors) improved TAC, as measured by ABTS and DPPH, compared to these drugs alone or insulin [37].

The FRAP method, based on electron transfer capacity, is frequently used for measuring TAC in healthy and diseased individuals due to its simplicity, sensitivity, and reliability [38,39,40,41,42]. Both decreased and increased FRAP have been reported in individuals with T2DM compared to controls, highlighting the complex nature of this parameter [39,40,41,42,43]. Higher FRAP values in the T2DMG may be due to interfering factors, in particular, increased levels of uric acid, a compound with strong reducing properties that may artificially increase FRAP results [42].

The three TAC assays used in this study assess distinct antioxidant mechanisms, which may explain the inconsistencies observed between DPPH, ABTS and FRAP. The first two methods primarily rely on hydrogen atom transfer and single electron transfer reactions to assess the radical scavenging activity of low-molecular-weight antioxidants, whereas FRAP quantifies the ferric-reducing ability of plasma. Moreover, TAC is a composite parameter and cannot distinguish whether lower antioxidant capacity in prediabetes reflects increased oxidative stress or reduced activity of enzymatic antioxidants.

### 4.2. Severity of Oxidative Stress in the Study Groups

In the context of this study, the values obtained using the ABTS and FRAP methods were highest in the group of people with T2DM, although differences between groups did not reach statistical significance. Interestingly, individuals with prediabetes had the lowest TAC in all analysed tests. Healthy individuals, on the other hand, achieved the highest values in the DPPH test, which may reflect the enhanced activity of lipophilic antioxidants.

The lower TAC values observed in individuals with prediabetes may reflect an early stage of redox homeostasis disturbances, in which the body’s compensatory mechanisms are not yet fully activated. In prediabetes, increased ROS production occurs due to insulin resistance and excess glucose, but the adaptive response of the antioxidant system (enzymatic and non-enzymatic) is relatively weakened during this period. As a result, TAC may decrease before the onset of full-blown T2DM [44,45,46].

The estimated effect sizes confirm that the observed differences between metabolic groups are not only statistically significant but also biologically significant. The highest effect size was observed for antioxidant capacity measured by ABTS (η^2^ ≈ 0.24), corresponding to a large effect size according to Cohen’s classification. This suggests that participants’ metabolic status has a significant influence on the overall free radical scavenging capacity measured by this method.

A moderate effect size was observed for DPPH parameters (η^2^ ≈ 0.14–0.15), suggesting that, despite significant differences between groups, the magnitude of change is smaller than for ABTS. The effect size for FRAP, which is close to moderate, also supports the concept of impaired antioxidant capacity in the context of type 2 diabetes and prediabetes.

Incorporating effect sizes into the interpretation of results is particularly important due to the lack of normality in the data and the differences between groups. The effect measures confirm that the differences may be clinically significant, indicating a need for further research on the role of antioxidant dysfunction in the pathogenesis of glycaemic disorders.

However, in individuals diagnosed with T2DM, the slightly higher TAC values observed (e.g., in FRAP and ABTS tests) may be due to several compensatory factors. First, chronic exposure to oxidative stress may induce the expression of endogenous antioxidant enzymes, which are part of the body’s adaptive response to persistent oxidative stress [47,48]. This phenomenon is referred to as adaptive antioxidant compensation, which involves a transient increase in TAC in response to oxidative stress. However, despite the increase in TAC, this does not necessarily translate into effective protection of cells from oxidative damage, suggesting that this mechanism is compensatory rather than preventative. [47,49,50]

Second, pharmacological therapy, especially metformin, may additionally modulate redox balance. Metformin exhibits antioxidant activity independent of its hypoglycaemic effect by activating the AMPK pathway, inhibiting mitochondrial complex I, and reducing ROS generation [51,,52]. Furthermore, most participants also had hypertension in addition to T2DM, and some antihypertensive drugs improve markers of oxidative stress, but the degree and reproducibility of this effect vary depending on the drug and patient population [53,54,55]. In this study, sensitivity analyses indicated that metformin use was associated with higher ABTS values, suggesting a potential enhancing effect of metformin on certain aspects of total antioxidant capacity. Other TAC measures (FRAP, DPPH) did not reach statistical significance, although moderate effect sizes suggest a trend. Interpretation is limited by the minimal number of participants receiving antidiabetic medications and by the small overall sample size.

### 4.3. The Effect of Diet on Oxidative Stress in the Study Groups

The analysis of the dietary patterns of the study groups (Table 3) revealed the lowest energy intake in individuals with T2DM compared to the other groups. This may be related to the desire to lose weight, as this group had the highest body weight and BMI compared to the others. Further results indicate a clear trend of declining dietary quality as carbohydrate metabolism disorders progress, from the CG through the PDG to individuals with T2DM. The PDG presented an intermediate nutritional profile between the T2DMG and CG, confirming that abnormalities in macro- and micronutrient intake occur even in the early stages of metabolic disorders.

The protein content of the diabetic patients’ diet exceeded the recommended level (22.2% of energy compared to the norm of 10–20%), confirming previous observations that increased protein intake can support glycaemic control and weight loss and prevent sarcopenia. At the same time, the lower proportion of plant protein in the T2DMG may indicate a less favourable protein source profile [56]. It is worth noting that the quality of dietary fats deteriorated as the disease progressed—individuals with T2DM consumed less MUFA and PUFA, while healthy individuals had a higher intake of unsaturated fats, including omega-3 and omega-6. Such differences may influence lipid profiles and cardiovascular risk [57]. T2DMG had the lowest intake of carbohydrates and simple sugars and the highest intake of fibre, which aligns with recommendations for individuals with T2DM [58]. However, all groups consumed an insufficient amount of fibre [59].

Micronutrient analysis revealed lower intakes of vitamins D, E, B2, and folic acid, as well as minerals (zinc, copper, and sodium) in the T2DMG and PDG compared to the CG. These values were often below the recommended values. Furthermore, too low B12 and phosphorus values were observed in T2DMG, and niacin and vitamin B6 in PDG. Low intakes of vitamin B1, B5, iron, calcium, magnesium, selenium, and potassium were observed in all groups. Each group had sufficient vitamin C intake [59]. Most study participants supplemented with 2000–4000 IU of vitamin D, while the remaining participants were advised to take supplements. The alarmingly low calcium intake is concerning, particularly in older individuals with a predisposition to osteoporosis [60]. Given that metformin lowers vitamin B12 levels, blood levels of this vitamin should be assessed in individuals with T2DM due to the potential for low intake of this micronutrient [61].

Spearman correlation analysis revealed significant associations between TAC and selected anthropometric parameters and nutrient intake; however, only some of them remained statistically significant after correction for multiple testing. DPPH values (both expressed as % and µmol TE/mL) were negatively correlated with body weight, BMI, and cholesterol intake. These results suggest that higher body weight and a higher BMI are associated with a reduced free radical scavenging capacity. This observation is consistent with previous reports that overweight and obese individuals had reduced serum TAC levels, which was explained by increased oxidative stress associated with chronic inflammation and insulin resistance [62]. The negative correlation between DPPH values and cholesterol intake may reflect the impact of a diet high in animal fat on the body’s oxidative status. Excessive cholesterol consumption can increase lipid peroxidation and free radical generation, resulting in a reduced serum antioxidant capacity [63,64]. Notably, after applying the Benjamini–Hochberg correction, the correlation with cholesterol intake did not remain statistically significant and therefore should be interpreted with caution.

Positive correlations were initially observed between vitamin B5, selenium, and dietary protein intake in the ABTS test. Vitamin B5 (pantothenic acid) plays a significant role in energy metabolism and may indirectly support the functioning of antioxidant systems by participating in the synthesis of coenzyme A and reducing oxidative stress in mitochondria [65]. Selenium, in turn, is a key trace element required for the proper activity of glutathione peroxidase (GPx) and other selenoproteins, which are responsible for neutralising lipid peroxides and protecting cell membranes from oxidative damage [66]. The positive correlation between ABTS and protein content may be due to the fact that a high-protein diet is often associated with an increased intake of sulphur-containing amino acids (e.g., cysteine), which are precursors of glutathione, one of the key endogenous antioxidants [67].

However, the negative correlations obtained using the ABTS test with omega-3, omega-6, MUFA, and PUFA content may seem counterintuitive, given the well-known anti-inflammatory and antioxidant properties of omega-3 fatty acids. However, fatty acids, especially polyunsaturated fatty acids, are particularly susceptible to lipid peroxidation, which, under conditions of increased oxidative stress, can lead to increased antioxidant consumption and decreased TAC [68,69]. Negative correlations between ABTS z values and phosphorus, calcium, and zinc may also reflect disturbances in mineral homeostasis in individuals with impaired carbohydrate metabolism [70,71,72]. Correlations between ABTS values and the intake of vitamin B5, omega-6 fatty acids, phosphorus, calcium, and zinc remained statistically significant after FDR correction, and the remaining values should be treated as a trend and interpreted with caution.

In the case of the FRAP test, initial negative correlations with omega-3 and omega-6 intake did not remain statistically significant after correction for multiple comparisons. Therefore, these findings should be interpreted with caution. Nonetheless, the direction of the associations is consistent with the notion that increased consumption of polyunsaturated fatty acids may elevate oxidative challenges due to their susceptibility to peroxidation, potentially increasing antioxidant utilisation [73].

In summary, the obtained results indicate that the body’s TAC is strongly associated with both anthropometric parameters and dietary quality. These results emphasise the importance of a balanced diet with adequate micronutrient content and a limited intake of saturated fats in modulating the oxidative-antioxidant balance in individuals with impaired carbohydrate metabolism. However, due to the combined correlations for the entire study population, it is essential to remember that this may mask intergroup differences; therefore, the obtained results should be interpreted with caution.

### 4.4. Limitations

This study represents a multidimensional approach to assessing TAC using three methods and provides a valuable analysis of nutritional status in individuals with T2DM and diabetes. The sample size was limited (*n* = 49) and heterogeneous, particularly in the prediabetes group, which reduces statistical power and increases the risk of random variability affecting the findings. In addition, the sex distribution across groups was unbalanced, with a predominance of female participants, and sex-stratified analyses were not feasible due to the small number of male participants. These factors may limit the generalisability of the results. Nevertheless, as already noted, this study was preliminary, and the findings should be interpreted with caution. For the same reason, regression models explained only a small portion of the variability in the dependent variables, suggesting the need for cautious interpretation of the results and confirmation of the observations in larger cohorts. The study design did not allow assessment of long-term glycaemic stability or clinical staging of diabetes severity. These factors may influence oxidative stress and should be addressed in future research. The nutritional analysis was based on data collected by a computer tool, which may not have a complete database of all vitamins and minerals (e.g., selenium and vitamin B5) in individual foods. Furthermore, the dietary interview is self-reported and subject to typical memory errors and underestimation of portion sizes. Data on supplementation and drug therapy, which could affect antioxidant status, are not available due to the ambiguity of survey responses and difficulties in their interpretation. No information was collected from participants regarding sleep duration and quality, which could influence oxidative stress. These data should be considered in future studies. Furthermore, the cross-sectional nature of the study precludes conclusions about the direction of causality between diet, metabolic parameters, and antioxidant activity.

PCA analysis was considered, but the KMO for the dietary and TAC variables was 0.504, and even lower for the other variables, indicating limited shared variance between these variables. Therefore, PCA was not an appropriate method for our dataset and was not used in further analysis.

Future studies should include larger and more balanced cohorts, incorporate mechanistic oxidative stress markers (e.g., GPx, SOD, catalase, lipid peroxidation products), and apply longitudinal or interventional designs to clarify causal relationships.

## 5. Conclusions

The study suggests significant differences in TAC between individuals with T2DM, prediabetes, and healthy controls. The lowest TAC values were observed in the prediabetes group, indicating a redox profile that differs from both healthy individuals and those with T2DM. Higher TAC values, as measured by ABTS and FRAP, in participants with T2DM may be influenced by treatment or metabolic adaptation, although causality cannot be established. Negative correlations were observed between DPPH-derived antioxidant activity and body weight and BMI, which were statistically significant. Associations with dietary cholesterol intake, FRAP values, and fatty acid intake were also noted, although these trends were not statistically significant after FDR correction. Positive correlations with ABTS were significant for vitamin B5, omega-6, phosphorus, calcium, and zinc. Other associations between ABTS values and dietary parameters were observed, but these non-significant trends should be treated with caution. Taken together, these results highlight that dietary composition and pharmacotherapy can significantly influence redox balance in metabolic disorders. Further studies with larger and more homogeneous cohorts are needed to confirm these preliminary findings and explore the potential for targeted nutritional interventions to improve antioxidant status in individuals with prediabetes and T2DM.

## Figures and Tables

**Table 1:** Characteristics and comparison of the studied groups.

Characteristics	T2DM Group (T2DMG) *n* = 19	Prediabetes Group (PDG) *n* = 12	Control Group (CG) *n* = 18	*p*-Value
Gender n (%)				0.6480
Female	17 (89)	9 (75)	15 (78)	
Male	2 (11)	3 (25)	4 (22)	
Age (years)	65.00 (61; 69.50) ^a^ 64.11 ± 7.33	63.50 (59.75; 65.75) ^a^ 63.25 ± 4.33	68.00 (63.50; 71) ^a^ 66.47 ± 4.95	0.1600
Body weight (kg)	82.00 (78.00; 91.00) ^b^ 83.47 ± 11.36	76.50 (65.03; 82.50) ^b^ 79.75 ± 23.03	63.00 (57.25; 70.00) ^a^ 63.97 ± 9.15	<0.0001
Height (m)	1.65 (1.60; 1.70) ^a^ 1.65 ± 0.07	1.62 (1.60; 1.69) ^a^ 1.64 ± 0.06	1.71 (1.69; 1.77) ^b^ 1.73 ± 0.10	0.0054
BMI (kg/m^2^)	30.10 (28; 34.15) ^b^ 30.65 ± 4.07	29.04 (25.35; 31.67) ^b^ 29.49 ± 6.74	21.60 (19.59; 23.02) ^a^ 21.37 ± 2.28	<0.0001
Place of residence n (%)				0.0004
City	9	7	18	
Village	10	5	0	
Fasting glucose (mg/dl)	132.00 (123.50; 161.50) 145.10 ± 32.00	113.50 (103.75; 120.5) 116.70 ± 17.40	– –	0.0021
HbA_1c_ (%)	6.29 (5.95; 7.42) 6.77 ± 1.13	5.58 (5.24; 5.83) 5.69 ± 0.61	– –	0.0013
Number of taken medications and supplements n (%)				0.0090
0	0 (0)	1 (8.30)	4 (22.22)	
(1–2]	6 (31.60)	4 (33.30)	12 (66.67)	
(2–4]	7 (36.80)	4 (33.30)	2 (11.11)	
(4–6]	4 (21.05)	1 (8.33)	0 (0)	
(6–9]	2 (10.50)	2 (16.70)	0 (0)	
Smoking cigarettes				0.5579
Yes	3	3	3	
No	16	9	16	
Alcohol consumption				0.1987
Once a week	2 (10.53)	3 (25.00)	2 (11.11)	
1–3 times a month	4 (21.05)	2 (16.67)	9 (50.00)	
No	13 (68.42)	7 (58.33)	7 (38.89)	
Physical activity				0.4710
No exercise	3 (15.79)	5 (44.67)	8 (44.44)	
Low (<150 min per week of moderate intensity)	7 (36.84)	4 (33.33)	3 (16.67)	
Moderate (150–300 min per week of moderate intensity)	5 (26.32)	2 (16.67)	3 (16.67)	
High (>300 min per week of moderate intensity)	4 (21.05)	1 (8.33)	4 (22.22)	

T2DM—Type 2 Diabetes, BMI—Body Mass Index, HbA_1c_—Glycated haemoglobin. Data are presented as Me (Q1; Q3) and X ± SD. Differences between groups were assessed using the Kruskal–Wallis test with Dunn’s post hoc analysis (Bonferroni correction). Qualitative were compared between groups using Fisher’s exact test. Groups marked with different letters differ statistically significantly (e.g., a, b) (*p* < 0.05), whereas groups marked with the same letter do not differ statistically (egg, a, a or b, b) (*p* > 0.05).

**Table 2:** Assessment of antioxidant activity using the DPPH (2,2-diphenyl-1-picrylhydrazyl radical), ABTS (2,2′-azino-bis(3-ethylbenzothiazoline-6-sulfonic acid)) and FRAP (Ferric Reducing Antioxidant Power) methods in the study groups.

Parameter	T2DM Group (T2DMG) n = 19	Prediabetes Group (PDG) n = 12	Control Group (CG) n = 19	*p*-Value	η^2^ (Effect Size)
**DPPH** **(%)**	8.82 (8.04; 12.60) ^a, b^ 10.41 ± 3.57	8.35 (6.73; 8.94) ^a^ 7.99 ± 1.65	10.65 (8.93; 12.43) ^b^ 10.78 ± 2.46	0.0404	0.14
			
(95% CI)	(8.69–12.13)	(6.94–9.04)	(9.60–11.96)		
DPPH (µmol TE/mL)	0.39 (0.34; 0.58) ^a, b^ 0.47 ± 0.18	0.36 (0.28; 0.39) ^a^ 0.34 ± 0.08	0.48 (0.39; 0.57) ^b^ 0.49 ± 0.13	0.0404	0.15
			
(95% CI)	(0.38–0.56)	(0.29–0.39)	(0.43–0.55)		
**ABTS (µmol TE/mL)**	3.52 (3.51; 3.6) ^b^ 3.53 ± 0.05	3.42 (3.32; 3.49) ^a^ 3.38 ± 0.16	3.44 (3.36; 3.53) ^a^ 3.43 ± 0.11	0.0080	0.24
			
(95% CI)	(3.51–3.55)	(3.28–3.48)	(3.38–3.48)		
**FRAP (mmol/L** **Fe ^2+^)**	0.96 (0.85; 1.21) ^b^ 1.06 ± 0.43	0.66 (0.59; 0.80) ^a^ 0.71 ± 0.22	0.95 (0.87; 1.08) ^b^ 1.06 ± 0.43	0.0049	0.13
			
(95% CI)	((0.85–1.27)	(0.57–0.85)	(0.85–1.27)		

T2DM—Type 2 Diabetes. Data are presented as Me (Q1; Q3) and X ± SD. Differences between groups were assessed using the Kruskal–Wallis test with Dunn’s post hoc analysis (Bonferroni correction). Groups marked with different letters differ statistically significantly (e.g., a, b) (*p* < 0.05), whereas groups marked with the same letter do not differ statistically (egg, a, a or b, b) (*p* > 0.05).

**Table 3:** Macro- and micronutrient intake between groups.

Nutrients	T2DM Group (T2DMG) *n* = 19	Prediabetes Group (PDG) *n* = 12	Control Group (CG) *n* = 19	*p*-Value
Calories (kcal/d)	1589.00 (1495.00; 1710.00) ^a^ 1625.26 ± 182.38	1889.50 (1599.75; 1996.00) ^a^ 1856.92 ± 297.32	1861.00 (1508.50; 2074.00) ^a^ 1866.16 ± 453.46	0.0625
Proteins (%)	22.20 (20.30; 23.70) ^b^ 21.73 ± 3.16	17.60 (16.30; 20.10) ^b^ 18.45 ± 2.70	14.60 (12.80; 16.20) ^a^ 14.56 ± 2.39	<0.0001
Fats (%)	31.00 (25.40; 34.45) ^a^ 31.95 ± 7.45	31.25 (28.65; 37.68) ^a^ 32.07 ± 9.00	35.40 (29.78; 37.40) ^a^ 34.36 ± 9.65	0.4338
Carbohydrates (%)	48.00 (44.30; 51.90) ^a^ 46.32 ± 7.84	51.30 (44.88; 54.15) ^a^ 49.49 ± 8.34	50.10 (47.80; 56.35) ^a^ 51.05 ± 10.12	0.2758
Proteins (g)	91.49 (76.82; 103.46) ^b^ 90.36 ± 16.15	82.13 (70.87; 105) ^a, b^ 87.02 ± 20.09	62.53 (54.81; 83.85) ^a^ 68.93 ± 23.12	0.0038
Animal protein (g)	46.46 (32.46; 61.97) ^a^ 45.62 ± 20.98	22.65 (16.28; 42.71) ^a^ 28.96 ± 20.79	39.98 (30.26; 58.06) ^a^ 42.68 ± 17.92	0.0822
Plant protein (g)	11.75 (10.22; 15.34) ^a^ 12.78 ± 3.64	14.57 (10.23; 16.51) ^a^ 14.57 ± 5.97	23.40 (21.42; 29.54) ^b^ 26.30 ± 9.41	<0.0001
Fats (g)	58.84 (48.12; 64.41) ^a^ 59.00 ± 13.93	69.66 (53.57; 80.40) ^a^ 67.41 ± 21.17	67.90 (59.29; 78.58) ^a^ 72.75 ± 32.29	0.1383
Saturated fatty acids (g)	17.74 (13.42; 19.51) ^a^ 17.62 ± 5.11	24.24 (18.28; 28.34) ^a, b^ 23.16 ± 7.47	26.14 (18.16; 32.14) ^b^ 26.03 ± 10.20	0.0066
Monounsaturated fatty acids (g)	14.20 (11.15; 21.12) ^a^ 15.79 ± 7.23	13.49 (10.74; 18.91) ^a^ 13.80 ± 6.50	25.99 (20.99; 35.66) ^b^ 30.33 ± 16.46	0.0001
Polyunsaturated fatty acids (g)	4.76 (3.46; 5.38) ^a^ 4.70 ± 1.99	4.02 (2.58; 6.36) ^a^ 4.63 ± 2.70	9.40 (6.84; 12.7) ^b^ 11.49 ± 7.90	<0.0001
Omega-3 (g)	0.66 (0.41;0.99) ^a^ 0.72 ± 0.36	0.63 (0.24; 1.50) ^a, b^ 0.98 ± 0.98	1.27 (0.85; 1.82) ^b^ 1.70 ± 1.54	0.0070
Omega-6 (g)	2.84 (2.31; 3.53) ^a^ 2.93 ± 1.08	1.69 (1.25; 3.16) ^a^ 2.25 ± 1.43	6.66 (4.71; 8.01) ^b^ 7.96 ± 6.34	<0.0001
Cholesterol (mg)	159.30 (125.52; 191.95) ^a^ 154.09 ± 51.31	153.95 (71.64; 211.69) ^a^ 161.14 ± 111.88	238.20 (174.83; 318.59) ^b^ 268.83 ± 141.00	0.0075
Carbohydrates (g)	195.15 (175.99; 211.99) ^a^ 192.91 ± 37.98	227.36 (205.89; 243.63) ^a, b^ 229.72 ± 44.66	233.45 (213.23; 274.51) ^b^ 249.54 ± 65.73	0.0033
Sugars (g)	33.29 (26.07; 39.23) ^a^ 33.10 ± 11.60	66.70 (47.6; 78.48) ^b^ 62.83 ± 28.56	49.77 (36.01; 65.67) ^b^ 50.39 ± 22.79	0.0032
Fibre (g)	19.70 (15.04; 25.72) ^a^ 20.36 ± 7.06	16.48 (15.10; 17.89) ^a^ 16.95 ± 4.38	18.03 (12.95; 22.37) ^a^ 17.78 ± 6.27	0.4090
Vitamin A (mg)	0.83 (0.43; 1.16) ^a^ 0.93 ± 0.72	0.77 (0.60; 0.92) ^a^ 0.84 ± 0.49	0.83 (0.47; 1,16) ^a^ 0.92 ± 0.67	0.9874
Vitamin B1 (mg)	0.62 (0.53; 0.86) ^a^ 0.78 ± 0.42	0.68 (0.46; 0.95) ^a^ 0.78 ± 0.41	0.78 (0.70; 0.91) ^a^ 0.91 ± 0.40	0.2353
Vitamin B2 (mg)	0.93 (0.70; 1.12) ^a^ 0.94 ± 0.30	0.99 (0.74;1.28) ^a, b^ 1.04 ± 0.48	1.28 (1.09; 1.5) ^b^ 1.38 ± 0.52	0.0103
Vitamin B5 (mg)	0.50 (0.27; 0.64) ^b^ 0.48 ± 0.26	0.21 (0.12; 0.68) ^b^ 0.56 ± 0.75	0 (0; 0.03) ^a^ 0.11 ± 0.25	<0.0001
Vitamin B6 (mg)	1.91 (1.69; 2.34) ^a^ 2.03 ± 0.62	1.22 (1.06; 1.88) ^a^ 1.48 ± 0.70	1.37 (1.23; 1.84) ^a^ 1.57 ± 0.71	0.0270
Vitamin B12 (μg)	1.37 (0.87; 1.86) ^a^ 1.75 ± 1.70	2.07 (1.17; 3.08) ^a, b^ 3.30 ± 4.23	2.75 (1.78; 3.26) ^b^ 2.85 ± 1.36	0.0099
Folic acid (mg)	0.24 (0.19; 0.29) ^a^ 0.24 ± 0.07	0.18 (0.13; 0.25) ^a^ 0.24 ± 0.22	0.26 (0.20; 0.29) ^a^ 0.28 ± 0.13	0.1430
Vitamin C (mg)	147.92 (114.13; 203.01) ^b^ 171.91 ± 96.73	120.18 (67.56; 156.80) ^a, b^ 129.66 ± 105.40	84.47 (43.69; 97.87) ^a^ 84.95 ± 57.90	0.0039
Vitamin D (μg)	0.58 (0.50; 1.34) ^a^ 1.84 ± 4.60	1.34 (0.63; 2.06) ^a, b^ 3.62 ± 6.41	1.35 (0.99; 1.94) ^b^ 1.79 ± 1.89	0.0301
Vitamin E (mg)	7.90 (6.19; 9.23) ^a^ 7.91 ± 2.58	6.43 (4.13; 7.78) ^a^ 6.78 ± 3.84	8.60 (7.64; 12.36) ^a^ 9.80 ± 4.93	0.0923
Niacin (mg)	17.48 (10.52; 18.52) ^a^ 15.59 ± 6.92	9.99 (5.40; 16.54) ^a^ 10.64 ± 6.22	12.22 (10.79; 19.73) ^a^ 14.97 ± 6.85	0.0940
Zinc (mg)	5.41 (4.22; 6.07) ^a^ 5.43 ± 2.08	6.25 (4.84; 7.61) ^a, b^ 6.21 ± 2.93	8.11 (6.22; 9.71) ^b^ 8.48 ± 2.87	0.0014
Phosphorus (mg)	649.27 (563.53; 770.75) ^a^ 649.83 ± 179.91	766.64 (523.91; 900.42) ^a^ 723.18 ± 378.45	1062.65 (912.25; 1382.38) ^b^ 1129.88 ± 347.94	0.0001
Magnesium (mg)	207.82 (159.40; 237.25) ^a, b^ 205.34 ± 65.39	165.95 (145.40; 186.18) ^a^ 172.53 ± 60.14	265.05 (216.46; 337.67) ^b^ 269.62 ± 91.34	0.0033
Copper (mg)	0.76 (0.66; 0.85) ^a, b^ 0.77 ± 0.22	0.64 (0.51; 0.75) ^a^ 0.64 ± 0.26	0.99 (0.80; 1.19) ^b^ 0.98 ± 0.33	0.0066
Potassium (mg)	2809.01 (2562.68; 3182.80) ^a^ 2895.48 ± 513.58	2146.09 (1677.93; 2702.03) ^a^ 2257.37 ± 874.09	2489.97 (2184.23; 2938.50) ^a^ 2620.84 ± 853.64	0.0580
Selenium (μg)	7.07 (2.18; 8.04) ^b^ 6.16 ± 4.60	2.69 (0.87; 8.82) ^b^ 7.40 ± 9.54	0 (0; 1.36) ^a^ 1.56 ± 2.92	0.0001
Sodium (mg)	1095.10 (633.61; 1312.86) ^a^ 1488.56 ± 1623.37	1064.11 (756.43; 1511.96) ^a^ 1169.68 ± 631.91	1769.47 (1465.59; 2013.60) ^b^ 2153.24 ± 1479.31	0.0016
Calcium (mg)	385.38 (308.21; 453.87) ^a^ 389.05 ± 178.00	544.99 (143.63; 819.9) ^a, b^ 533.63 ± 388.52	747.55 (474.91; 888.88) ^b^ 724.85 ± 267.74	0.0025
Iron (mg)	6.46 (5.76; 8.81) ^a^ 6.79 ± 1.70	5.70 (4.28; 7.7) ^a^ 6.01 ± 2.15	9.60 (7.51; 11.99) ^b^ 10.20 ± 3.78	0.0005

T2DM—Type 2 Diabetes. Data are presented as Me (Q1; Q3) and X ± SD. Differences between groups were assessed using the Kruskal–Wallis test with Dunn’s post hoc analysis (Bonferroni correction). Groups marked with different letters differ statistically significantly (e.g., a, b) (*p* < 0.05), whereas groups marked with the same letter do not differ statistically (e.g., a, a or b, b) (*p* > 0.05).

**Table 4:** Monotonic relationships between antioxidant activity (DPPH (2,2-diphenyl-1-picrylhydrazyl radical), ABTS (2,2′-azino-bis(3-ethylbenzothiazoline-6-sulfonic acid)) and FRAP (Ferric Reducing Antioxidant Power) and anthropometric, dietary and biochemical parameters.

Parameter	r Spearman	Two-Sided *p*-Value	q-Values (FDR)
DPPH (%) vs. Body weight (kg)	−0.40	0.0095	N/A
DPPH (%) vs. BMI (kg/m^2^)	−0.33	0.0381	N/A
DPPH (%) vs. Cholesterol (g)	−0.40	0.0261	0.3332
DPPH (µmol TE/mL) vs. Body weight (kg)	−0.40	0.0095	N/A
DPPH (µmol TE/mL) vs. BMI (kg/m^2^)	−0.33	0.0381	N/A
DPPH (µmol TE/mL) vs. Cholesterol (g)	−0.40	0.0261	0.3332
ABTS (µmol TE/mL) vs. Vitamin B5 (μg)	0.53	0.0004	0.0135
ABTS (µmol TE/mL) vs. Selenium (μg)	0.38	0.0153	0.0601
ABTS (µmol TE/mL) vs. Protein (%)	0.34	0.0305	0.0807
ABTS (µmol TE/mL) vs. Omega-3 (g)	−0.37	0.0163	0.0601
ABTS (µmol TE/mL) vs. Omega-6 (g)	−0.44	0.0042	0.0220
ABTS (µmol TE/mL) vs. Monounsaturated fatty acids (g)	−0.35	0.0229	0.0736
ABTS (µmol TE/mL) vs. Polyunsaturated fatty acids (g)	−0.35	0.0239	0.0736
ABTS (µmol TE/mL) vs. Phosphorus (mg)	−0.50	0.0009	0.0328
ABTS (µmol TE/mL) vs. Calcium (mg)	−0.46	0.0024	0.0176
ABTS (µmol TE/mL) vs. Zinc (mg)	−0.49	0.0012	0.0138
FRAP (mmol/l Fe^2+^) vs. Omega-3 (g)	−0.35	0.0130	0.3657
FRAP (mmol/l Fe^2+^) vs. Omega-6 (g)	−0.33	0.0198	0.3657

BMI—Body Mass Index, N/A—Not applicable. Spearman correlation coefficient (r) and two-sided *p* values for each pair of variables. *p* values < 0.05 were considered statistically significant. q-values were calculated using the Benjamini–Hochberg procedure to correct for multiple comparisons. Only correlations with q < 0.05 are considered statistically significant, while other observed associations are reported as non-significant trends.

## Data Availability

The datasets presented in this article are not readily available because the data are part of an ongoing study. Requests to access the datasets should be directed to the corresponding author.

## Abbreviations

The following abbreviations are used in this manuscript:

ABTS	2,2′-azino-bis(3-ethylbenzothiazoline-6-sulfonic acid)
AMPK	AMP-activated protein kinase
BMI	Body Mass Index
CG	Control Group
DPPH	2,2-diphenyl-1-picrylhydrazyl
FDR	False discovery rate
FRAP	Ferric Ion Reducing Antioxidant Parameter
GPx	Glutathione peroxidase
HbA_1c_	Glycated haemoglobin
IQR	Interquartile range
Me	Median
MUFA	Monounsaturated fatty acids
Nrf2	The nuclear factor erythroid 2-related factor 2
PDG	Prediabetes Group
PUFA	Polyunsaturated fatty acids
Q	Quartile
ROS	Reactive oxygen species
SD	Standard Deviation
T2DM	Type 2 Diabetes Mellitus
T2DMG	Type 2 Diabetes Mellitus Group
TAC	Total Antioxidant Capacity
TE	Trolox Equivalent
TEAC	Trolox equivalent antioxidant capacity
WHO	World Health Organisation
X	Mean
